# Analyzing synthesis routes for BaCuPO_4_: implications for hydrogen evolution and supercapattery performance†

**DOI:** 10.1039/d3ra07596f

**Published:** 2023-12-05

**Authors:** Sarfraz Ali, Haseebul Hassan, Muhammad Waqas Iqbal, Amir Muhammad Afzal, Mohammed A. Amin, A. Alhadrami, Nawal D. Alqarni, Ehtisham Umar

**Affiliations:** a Department of Physics, Riphah International University, Campus Lahore Pakistan; b Department of Chemistry, College of Science, Taif University P.O. Box 11099 Taif Saudi Arabia; c Department of Chemistry, College of Science, University of Bisha Bisha 61922 Saudi Arabia; d Department of Physics, Government College University Lahore 54000 Punjab Pakistan waqas.iqbal@riphah.edu.pk

## Abstract

In recent years, energy storage and conversion tools have evolved significantly in response to rising energy demands. Owing to their large surface area, superior electric and chemical stabilities, and thermal conductivities, barium copper phosphate (BaCuPO_4_) materials are promising electrode materials for electrochemical energy storage systems. In this study, the synthesis of nanostructures (NSs) using hydrothermal and chemical precipitation methods and exploring the electrochemical characteristics of BaCuPO_4_ in asymmetric supercapacitors provides a comparative investigation. Systematic characterization shows that nanomaterials prepared by applying the hydrothermal method have a more crystalline and large surface area than chemical precipitation. In the three cell arrangements, the hydrothermally prepared BaCuPO_4_ NSs delivered a high specific capacity (764.4 C g^−1^) compared to the chemical precipitation route (660 C g^−1^). Additionally, the supercapattery associated with the two electrode assemblages delivers an optimum specific capacity of 77 C g^−1^. The energy and power density of BaCuPO_4_//AC NSs were 52.13 W h kg^−1^ and 950 W kg^−1^, respectively. A durability test was also performed with BaCuPO_4_//AC NSs for 5000 consecutive cycles. Further, the coulombic efficiency and capacity retention of BaCuPO_4_//AC after 5000 cycles were 81% and 92%, respectively. Bimetallic phosphate is comparatively suggested for future perspectives towards HER to overcome the performance of single metal phosphate materials. This is the first approach, we are aware of, for investigating the electrochemical behavior of BaCuPO_4_, and our results suggest that it may be useful as an electrode material in electrochemical systems requiring high energy and rate capabilities.

## Abstract 2

In recent years, energy storage and conversion tools have evolved significantly in response to rising energy demands.

## Graphical abstract

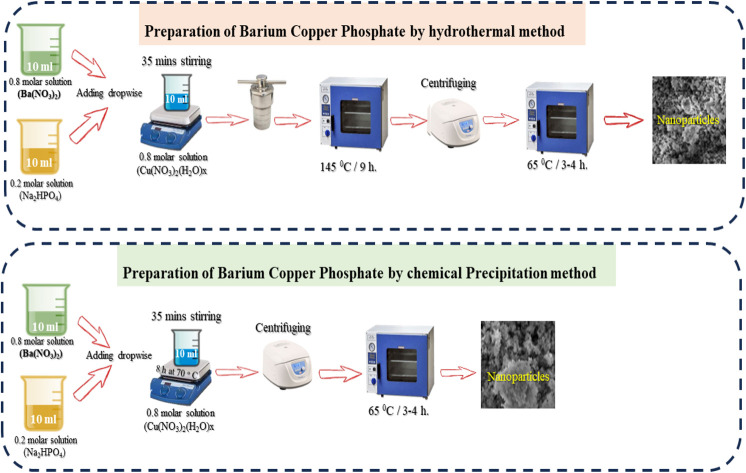

## 1. Introduction

Pursuing sustainable and commercially viable energy conversion and storage methods has emerged as a pressing difficulty encountered in energy research owing to the reduction of fossil fuels. As viable energy resources that may be utilized extensively rather than occupying the demerits of environmental pollution, these resources will soon be depleted.^1,2^ The main research interest is to develop a clean and inexpensive path towards energy storage concerning satisfaction with the worldwide immense requirements.^3^ Batteries and supercapacitors are suggested to be more suitable promising candidates that may resolve these problems on a priority basis because of their appropriate characteristics and merits. Batteries possessing extraordinary volumetric and gravimetric energy densities compared with alternative energy storage strategies have made them capable of compactible electric fields.

However, they become inefficient in vast potential applications due to their dependency on temperature and lower power density.^4–6^ On the contrary, supercapacitors may be categorized into pseudocapacitors and electric double-layer capacitors (EDLC), which are well-equipped with storage mechanisms. Function with the same analogy of mechanism in the form of rechargeable batteries and the similarity in fabrication and architecture of both. Further, they depend on two conductive electrodes implanted into electrolytes containing free charge carriers.^7–9^ Further, captivating consideration, active charge–discharge capabilities, piousness-proof behavior and lower conservation cost prioritize them over other alternative storage expertise.^10–12^ However, the minor energy density belonging to supercapacitors disqualifies their role in versatile applications in the modern era. To date, remarkable efforts have been made to enhance the capacity of energy storage technologies, but their success has been limited.^13^

Now, the merging of EDLC with battery technology introduces the emergence of a new technology known as supercapattery.^14^ Advantageously, supercapattery possesses energy storage with elevating power/energy density, efficient charge–discharge capability, prolonged cycles and flexible temperature range that may envelop both supercapacitors and batteries with combined properties. Additionally, supercapattery technology may aqueduct the energy and power density gap between supercapacitors and batteries.^15,16^ The main advantage of the architecture of composite devices is achieving optimal energy originating from battery-nature material along with the interaction of supercapacitor nature materials to deliver high power capability. Because of the combined technology, supercapattery hybrid devices may dominantly enhance the cell potential and broaden its lifetime.^17,18^

Recently, research struggle has contributed a significant part to developing electrode-nature materials owing to supercapacitors, including carbon-derived ingredients, metallic oxides, conductive-nature polymers, and newly introduced material of metal–organic framework (MOF).^18–21^ Among transition metal oxides (TMO), positive electrode cobalt-based materials have been extensively studied for supercapattery technology.^22,23^ Furthermore, phosphate-based materials have exhibited better commercial performance regarding battery fabrication.^24^ In addition, phosphate-based hybrid devices have offered remarkable specific energy but not a reasonable retention rate.^25,26^

Therefore, before discussing the comparison between the desired synthesis methods, the merits and demerits of adopting synthesis approaches should be overviewed owing to different nanomaterials, particularly phosphate-based materials, which have been fabricated by employing considerable strategies. Recently, numerous electrode-nature materials in supercapacitor applications have been utilized, such as metal oxides (Co_3_O_4_, MnO_2_, and NiO, *etc.*),^27–29^ and also hydroxides, such as Co(OH)_2_) and (Ni(OH)_2_.^30^ Zhang *et al.* fabricated CuO architecture *via* a surfactant-aided wet chemical approach, and the specific capacitance of 88.5 F g^−1^ at 2 A g^−1^ was achieved.^31^ Moreover, Ji *et al.* prepared mesoporous NiO nanosheets by employing a hydrothermal strategy, in which the synthesized material showed a specific capacitance of 674.2 F g^−1^ allied with a particular capacity of 84.274 mA g^−1^ at 1 A g^−1^.^32^ Another study on cobalt phosphate nano-/microstructure fabrication by employing a hydrothermal strategy in supercapattery electrode material showed that the device effectively functions inside the human body for 15–20 years. In this study, the main focus describes the synthetic nanoflakes/nanoflowers owing to cobalt phosphate that derived supercapattery devices encompassing their mechanism, structure and electrochemical properties by showing excellent performance because it could replace the battery device with the developed self-rechargeable pacemaker as an alternative source.^33^ Moreover, battery-nature electrodes have extensively enhanced the specific capacitance and showed the desired energy density for supercapacitors caused by rapid and reversible processes when linked with PS electrodes. Various cobalt/nickel composite-based devices are mainly utilized as supercapacitors.^34,35^ Metal phosphates are suggested as the most promising materials for catalysts and energy-reaping implementations owing to their effective potential windows, fine-layered construction, better reversible mechanism, higher conducting ability, and lower cost. Pang *et al.* fabricated amorphous-nickel-phosphate nanorods for flexible supercapacitor applications, which exhibited a specific capacitance of 145.8 mA h g^−1^ with 0.5 A g^−1^.^36^ Wang *et al.* fabricated Ba_*x*_Ni_3−*x*_(PO_4_)_2_ grain-based-species *via* exchange reaction route, which showed a specific capacitance of 1058 F g^−1^ with a current density of 0.5 A g^−1^.^37^ Na-doped Ni_2_P_2_O_7_ hexagonal-tablets have been fabricated by following a calcination route, which has offered a great specific capacitance of 557.7 F g^−1^ at 1.2 A g^−1^. Additionally, Ni–Co phosphate amorphous nature microplates have offered a large specific capacitance of 1259 F g^−1^ for current density at 1.5 A g^−1^.^38^

Herein, a comparative study of two synthesis methods (hydrothermal and chemical precipitation) was discussed to prove which method would be versatile from a synthetic perspective. The main focus of this study is on comparatively electrochemical measurements of barium copper phosphate (BaCuPO_4_) material following two parallel synthesis strategies (hydrothermal and chemical precipitation) for supercapattery implementation. X-ray diffractometry and scanning electron microscopy investigated the crystallinity and morphological structure of the synthesized material (BaCuPO_4_). In contrast, CV and GCD results were used to elaborate the electrochemical behavior of BaCuPO_4_ synthesized through hydrothermal and chemical precipitation methods. Supercapattery was also fabricated through BaCuPO_4_ and activated carbon. Electrochemical studies and stability tests were also performed with this supercapattery (BaCuPO_4_//AC). The hydrogen evolution reactions (HER) tests were also performed to confirm the promising candidate for bimetal phosphate//activated carbon materials for future hydrogen production. In this study, using BaCuPO_4_ as a fundamental building block for fabricated supercapacitor electrodes is a novel approach. This study investigates the synthesis and characterization of BaCuPO_4_ NSs as supercapacitor electrodes, revealing their tremendous potential for SC applications.

## 2. Experimental section

### 2.1 Precursor materials

First, potassium hydroxide (KOH) was acquired from Sigma Aldrich. Hydrochloric acid (HCl), *N*-methyl 2-pyrrolidone (NMP), carbon black (CB), *N*-polyvinylidene-fluoride (PVDF), copper(ii) nitrate hydrate (Cu(NO_3_)_2_(H_2_O)_*x*_, and barium nitrate Ba(NO_3_)_2_ were obtained from Korean DUKSAN, and sodium hydrogen phosphate (Na_2_HPO_4_) was obtained from SAMCHUN. Ni foam was acquired from Urich Technology Malaysia. All the precursor materials purchased were utilized without further purification. Further, Hg/HgO and platinum wire were obtained from ALS Co. Ltd in Japan and utilized as counter and reference electrodes, respectively, during the assembly of a three-electrode construction.

### 2.2 Synthesis of materials

Facial synthesis techniques were adopted to synthesize BaCuPO_4_ NSs through hydrothermal and chemical precipitation methods, which were comparatively simple and easy to synthesize using other sol–gel and sonochemical synthesis methods.^39^

#### 2.2.1 Preparation of BaCuPO_4_ NSs using the hydrothermal method

**Fig. 1:**
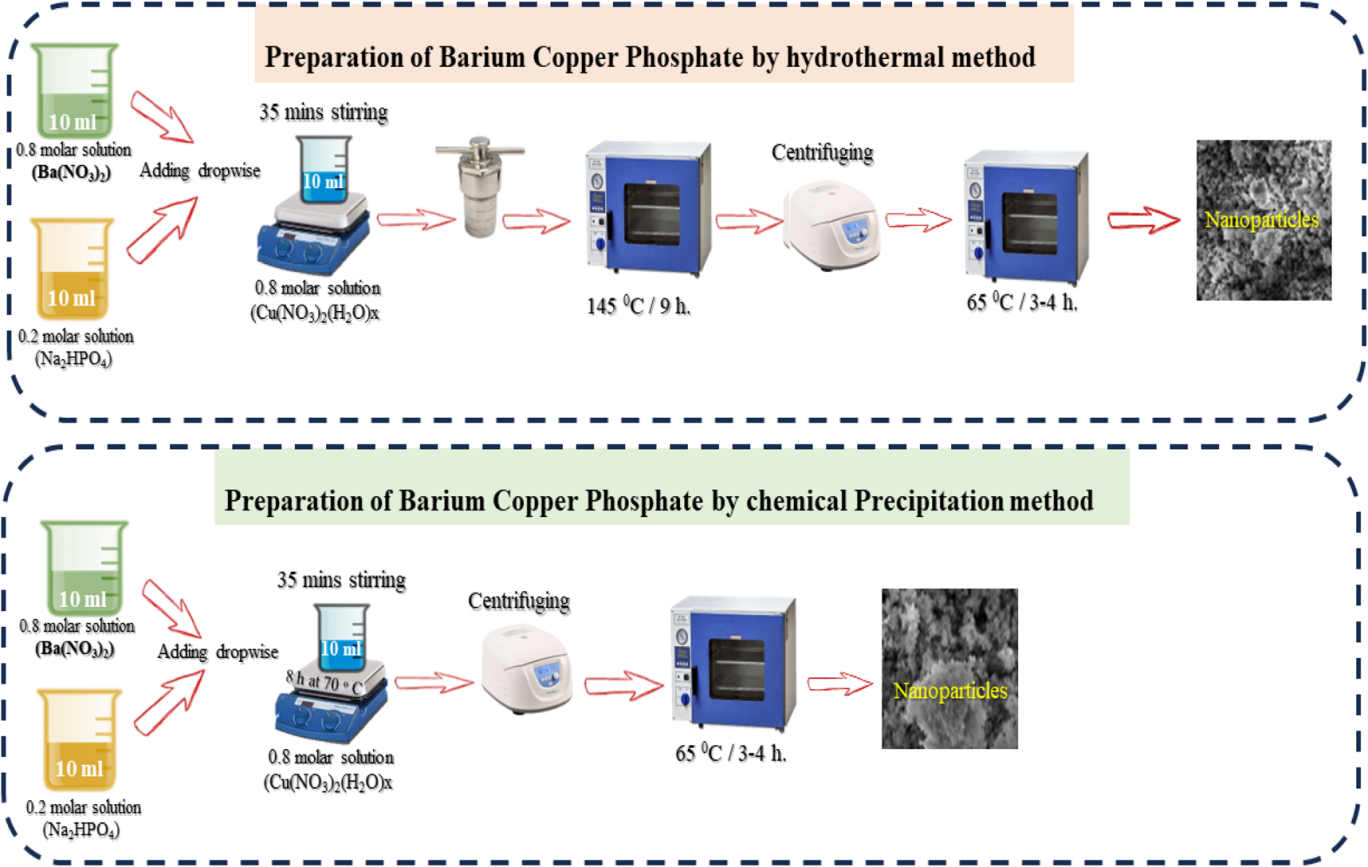
Schematic of hydrothermal and chemical precipitation synthesis of BaCuPO_4_NSs.

The hydrothermal method achieves the targeted crystalline NSs in a high-temperature environment.^39^ The 0.8 M solutions were prepared by adding 2.816 g of Cu(NO_3_)_2_·(H_2_O)_*x*_ into 10 ml deionized (DI) water under vigorous stirring. The 0.8 M Ba(NO_3_)_2_ solution was synthesized through deliquesce 2.145 g of Ba(NO_3_)_2_ into 10 ml of DI water. After that, 0.30 g sodium phosphate dibasic anhydrous Cu(NO_3_)_2_ was mixed into 10 ml of DI water to prepare a 0.2 M solution. Both solutions Ba(NO_3_)_2_ and Na_2_(HPO_4_) were then transferred into Cu(NO_3_)_2_·(H_2_O)_*x*_ solution. After 35 min of stirring, the prepared solution was successfully shifted into a Teflon-based autoclave at 145 °C for 9 h. The greenish-colored precipitating solution was centrifuged with DI water and ethanol several times to remove impurities. The collected sediments were dried in an oven at 65 °C for 4 h (Fig. 1).

#### 2.2.2 Preparation of BaCuPO_4_ NSs through the chemical precipitation method

Another chemical precipitation strategy was also employed to synthesize BaCuPO_4_ for a comparative study. The chemical precipitation strategy was suggested as the simplest and time-saving method to synthesize the required materials. The same amounts of all precursors were used to synthesize BaCuPO_4_ through the chemical precipitation method. The only difference is that the chemical precipitation method did not transfer the materials into the autoclave. The combined solution (Cu(NO_3_)_2_·(H_2_O)_*x*_, Ba(NO_3_)_2_ and Na_2_HPO_4_) were stirred for 8 h at 70 °C. After that, the colloidal solution containing synthetic nanoparticles was successfully centrifuged to eliminate impurities (Fig. 1).

## 3. Working electrode preparation and electrochemical activity

Herein, the working electrode fabrication was initiated by a Ni-foam current collector, while the substrate was washed with HCl, water and ethanol for the deposition of the available material. NMP is a solvent for preparing homogenous slurry involving 80% active electrode materials (BaCuPO_4_) with 10% PVDF and 10% carbon black. The mixing species are magnetically stirred for 6 h to collect suitable slurry. The slurry is homogeneously coated on a 1 × 1 cm^2^ surface area of Ni foam. Then, the created working electrode is subjected to a temperature of 55 °C for drying before proceeding with electrochemical measurements. Electrochemical characterization was accomplished using a 1 M KOH solution as the electrolyte, with Hg/HgO as the reference electrode and the counter electrode as a platinum wire.

## 4. Results and discussion section

### 4.1 X-ray diffraction (XRD) results

**Fig. 2:**
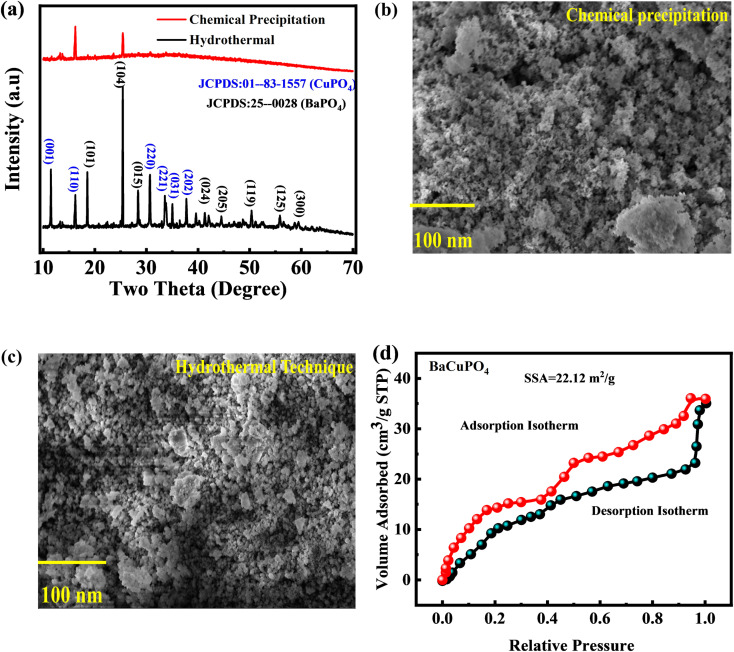
(a) XRD pattern revealing individually chemical precipitation and hydrothermal routes, (b) SEM micrograph of chemical precipitation prepared NSs, (c) SEM micrograph of hydrothermal prepared NSs, and (d) BET measurements for adsorption–desorption isotherms of synthesized BaCuPO_4_ NSs.

XRD analysis was used to investigate the crystallinity and phase impurities of the prepared sample BaCuPO_4_ NSs (Fig. 2a). Crystallite size was calculated using the Debye Scherer equation:1
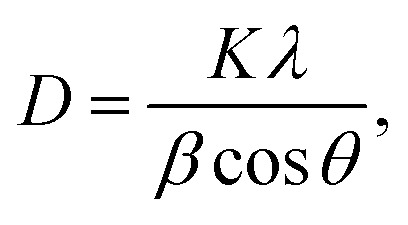
where *D* denotes crystallite size, the wavelength of the X-ray is *λ*, and *β* denotes the FWHM value. The synthetic crystallite sizes estimated BaCuPO_4_ through hydrothermal and chemical precipitation techniques NSs were 56 nm and 50 nm, respectively. Diffraction peaks at (101), (104), (015), (024), (205), (119), (125), and (300) planes were evident with the appearance of crystalline BaPO_4_ species corresponding to JCPDS card no. 25-0028.^40^ Moreover, owing to the formation of CuPO_4_, significant peaks were revealed at (001), (110), (220), (221), (031), and (202), which were confirmed by JCPDS-01-083-1557.^41^ The deep analysis of XRD patterns reveals that the hydrothermal outcomes of intense peaks offer higher crystallinity in the as-prepared material. Moreover, owing to thoroughly narrowed and sharp peaks, the behavior of the fabricated devices is more closely detected as supercapattery performance. However, the diffraction patterns of the XRD results observed *via* the chemical precipitation technique were close to those of a very weak crystalline material, probably amorphous in nature, as revealed by the appearance of thoroughly prominent blurred peaks. The XRD results were also subsequently confirmed with a comparative study of the SEM analysis of both routes as well as their electrochemical measurements.

### 4.2 Scanning electron microscopy (SEM)

The microstructural and surface morphological analyses of the synthesized NSs through different routes (hydrothermal and chemical precipitation) were analyzed through SEM micrographs (Fig. 2b and c). The hydrothermally prepared NS SEM micrograph shows more prominent and clear results than the chemical precipitation SEM NSs, which are well synchronized with the XRD peak intensity directly related to the crystallinity of the NSs. The XRD results of the hydrothermally prepared NSs have a high peak intensity, indicating that they have higher crystallinity than the XRD results of chemically precipitated NSs. SEM images of NSs show that agglomerations of irregular-sized nanoparticles provide more active sights and networks of nanofibers, leading to the availability of change carriers and increasing the efficiency of energy storage devices.

### 4.3 X-ray photoelectron spectroscopy (XPS)

**Fig. 3:**
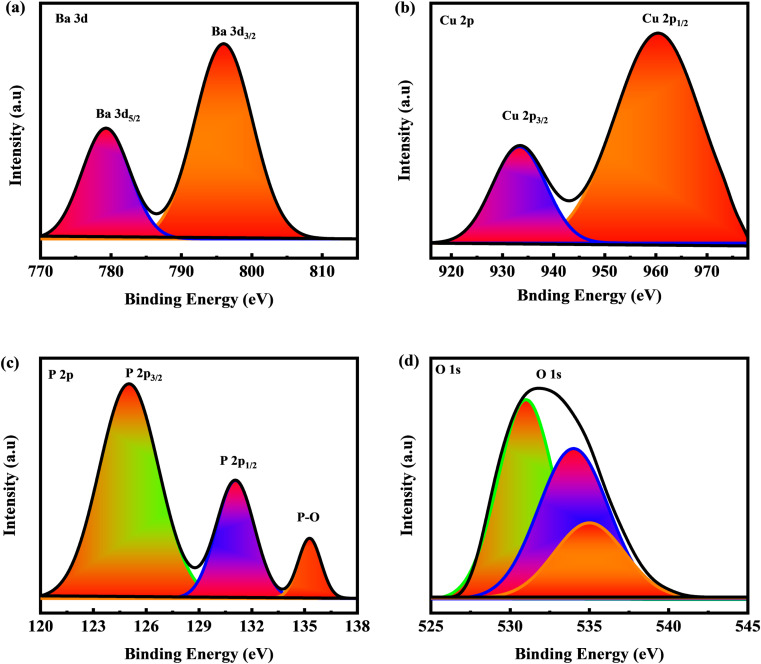
XPS spectra of Ba 3d, Cu 2p, P 2p, and O 1s, respectively of BaCuPO_4_ NSs.

XPS was utilized to determine the oxidation states and surface electronic structure of the synthesized-doped free and doped NSs (Fig. 3). The prominent XPS spectra of the Ba 3d core level discovered a pair of peaks (Fig. 3a) at binding energies (BEs) of 778.2 eV and 795.3 eV, correlated with Ba 3d_5/2_ and Ba 3d_3/2_, respectively, belonging to the Ba^2+^ state.^42^ The deconvoluted XPS spectra of Cu 2p show a couple of peaks Cu 2p_3/2_ and Cu 2p_1/2_ at BE of 934.5 eV and 962.3 eV, respectively (Fig. 3b).^43^Fig. 3c depicts the two distinct spectra of P 2p_3/2_ and P 2p_1/2_ at BE of 124.5 eV and 131.2 eV, respectively, with an additional P–O spectrum located at 135.5 EeV.^44^ O 1s state data indicate consistent binding energy shifts, with dissociated oxygen species, adsorbed (OH·), and water molecules (H_2_O) at 530.6, 532.6, and 533.8 eV, respectively (Fig. 3d).^45^

### 4.4 BaCuPO_4_ adsorption–desorption measurements

It is observable that electrochemical performance may be affected by the structure and morphology of the synthesized BaCuPO_4_ NSs using different methods. Therefore, pore size/volume distribution and specific surface area are the parameters of N_2_ isothermal adsorption–desorption measurements and the BET method examination. The absorption hysteresis loop ranges from 0.1 to 1 at *P*/*P*_0_ (relative pressure), as shown in Fig. 2d, and the corresponding isotherm curve is proved as type-IV depending on the IUPAC classification standard. The large surface area provides attractive surface sites with promising charge transfer pathways, thereby allowing the rapid transportation of ions/electrons during electrochemical reactions and promoting excellent electrochemical properties. Hence, these findings support further electrochemical measurement studies.^46–48^ The hysteresis loop in this structure indicates a mesopore structure, which is responsible for the reduction in the diffusion distance attributed to electrolyte ions. Moreover, the reduced BET surface area of 22.12 m^2^ g^−1^ reveals the random distribution of the mesopores of Ba(NO_3_)_2_ and Cu(NO_3_)_2_ with Na_2_(HPO_4_) to form a uniform distribution of mesopores of the composite of BaCuPO_4_.^49^ The BET isotherm of BaPO_4_ and CuPO_4_ nanocomposites is shown in Fig. S1.† This would probably lead to highly improved properties of the electrochemical performance of supercapattery devices.

## 5. Electrochemical measurements

**Fig. 4:**
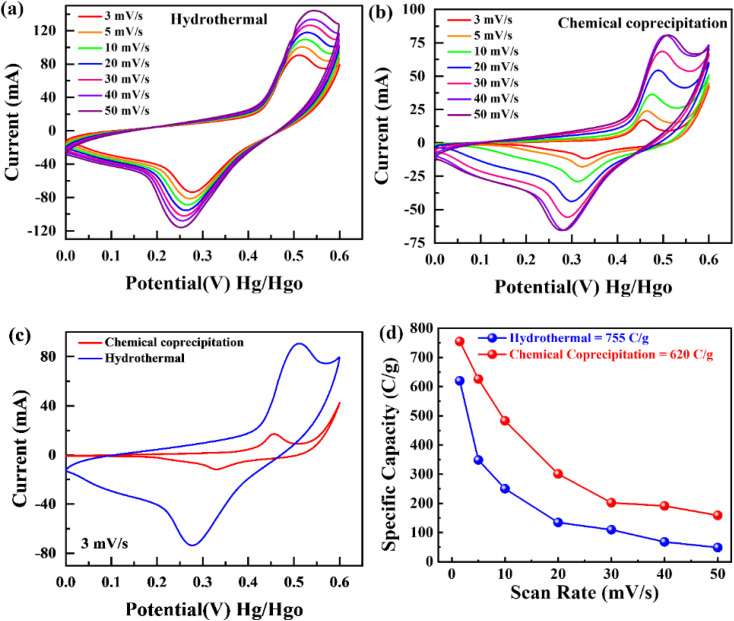
Cyclic voltammetry of BaCuPO_4_ at various scan rates: (a) hydrothermal prepared NSs, (b) chemical precipitation prepared NSs, (c) comparative study of two approaches at specific scan rate, and (d) specific capacity comparison between both strategies.

To examine the type of NSs and their intrinsic properties, cyclic voltammetry (CV) measurements were performed for the as-prepared BaCuPO_4_ by applying chemical precipitation and hydrothermal methods, as illustrated in Fig. 4a and b. For a comparative study, the CV of BaPO_4_ and CuPO_4_ nanocomposites at a 3 mV s^−1^ scan rate is depicted in Fig. S2.† In addition, CV plots for CuPO_4_ are drawn at scan rates of 3–50 mV s^−1^ in the corresponding potential window. Redox peak trends are observed rightward, corresponding to higher potential values with increased values of scan rate, as illustrated in Fig. 4a and b. Further, the observed prominent redox peaks are evident in the battery nature of the prepared nanomaterial caused by the strong interaction of electrode and hydroxide ions. The CV results clearly show that the crystalline material obtained by the hydrothermal strategy is strongly efficient in battery-type electrochemical performance. In addition, the comparatively CV curves demonstrated in Fig. 4c support the declaration of highly electrochemically efficient material prepared by the hydrothermal route. However, the specific capacity through a CV for different synthesis strategies is calculated using the following basic equation:2
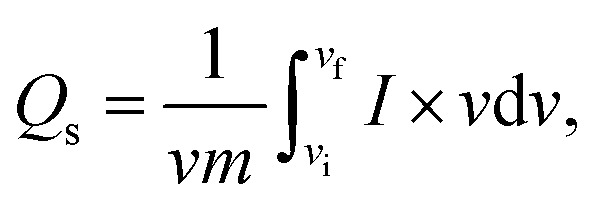
where *Q*_s_ denotes specific capacity (eqn (2)). The total integral part gives the area covered by the curve. Additionally, *v* denotes scan rate, and *m* represents active mass. As depicted in Fig. 4d, the specific capacity attributed to CV curves against various scan rates was comparatively analyzed for different strategies to strengthen the performance of hydrothermal, as in previous results. As a whole, the specific capacity curve always lies above the capacity curve of chemical precipitation, which repeatedly supports the better performance of the hydrothermal technique. Furthermore, as shown in Fig. 4d, the comparatively measuring value of the specific capacity (755 C g^−1^) is far larger than the chemical precipitation value (620 C g^−1^), which is evidence of betterment for the hydrothermal platform. Table 2 provides a comparative analysis of our Qs with those of previous studies.

**Fig. 5:**
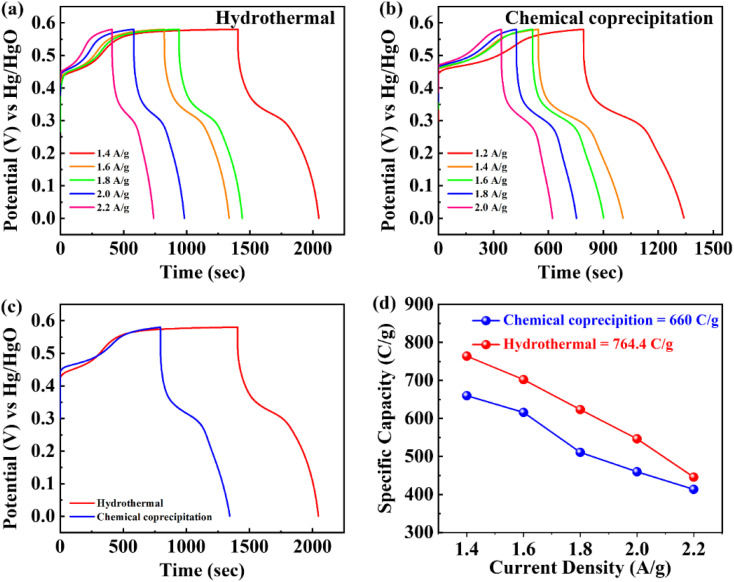
Galvanostatic charge–discharge of BaCuPO_4_ NSs (a) by hydrothermal and (b) by chemical precipitation. (c) Comparative study for both approaches and (d) specific capacity analysis for both strategies.

As depicted in Fig. 5a and b, the GCD curves of BaCuPO_4_ exhibit a non-linear trend that confirms redox reaction active sites over the electrode material surface, which is also characteristic of battery-grade materials and has been proven by CV measurements. For a comparative study, the GCD of BaPO_4_ and CuPO_4_ nanocomposites is depicted in Fig. S3.† When current densities are consecutively increased, the charge/discharge time responses decrease but with no shape change in curves, revealing the stable behavior of battery-graded synthesized material. The discharging time duration is caused by a delay owing to the interaction with the active electrode. As demonstrated in Fig. 5c, the comparative trend of the GCD curves reveals an efficient performance in charging/discharging time. Furthermore, specific capacity is computed from the GCD curves (Fig. 5d) using the following equation:3
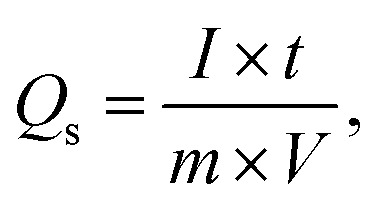
where *Q*_s_ represents specific capacity and *I* represents current. Moreover, denoted discharge interval time along with *m* representing active mass (eqn (3)). In addition, the optimum values of specific capacities from the GCD curves for hydrothermal and chemical precipitation approaches were evaluated as 764.4 C g^−1^ and 660 C g^−1^, associated with a current density of 1.2 A g^−1^ at the same level, respectively. Consequently, high crystalline and synergistically electrochemical characterized material, synthesized by hydrothermal strategy, proved more authentic for the fabrication of better electrochemical energy storage devices. Again, the hydrothermal strategy achieves better electrochemical performance in terms of greater rate capability. The specific capacity trending toward decreasing values with increasing current density is still favourable in hydrothermal results because of the short contact time. GCD analysis findings carried out at different current densities with constant potential are also strengthened by relative CV results (as already evident by improved electrochemical performance by hydrothermal route rather than chemical precipitation approach). Hence, both fundamental electrochemical characteristics CV and GCD strongly support hydrothermal measurements towards the same sample for both routes, but more novel for hydrothermal.

### 5.1 Supercapattery performance

**Fig. 6:**
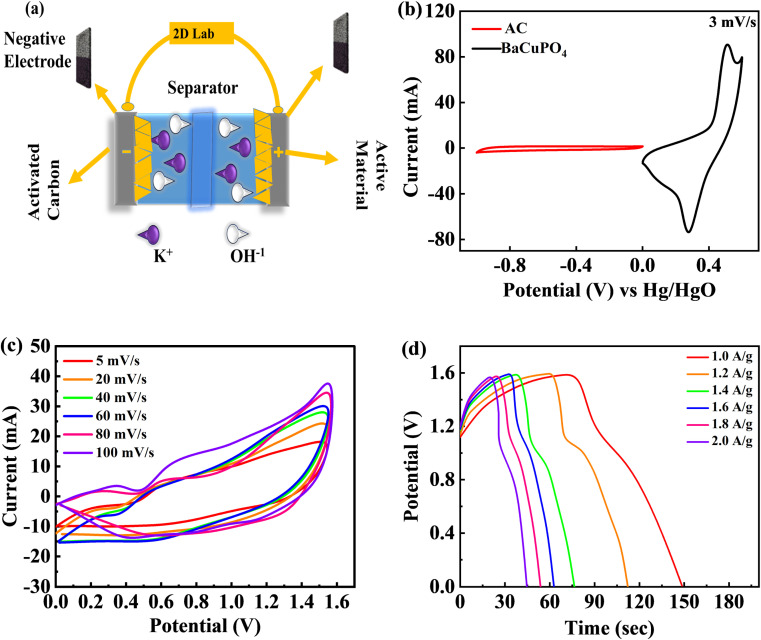
(a) Schematic diagram of supercapattery mechanism, (b) CV comparative analyses of cathode and anode for supercapattery device, (c) CV performance of supercapattery at 5–100 mV s^−1^ scan rates using novel hydrothermal approach, and (d) GCD of BaCuPO_4_//A.C. at various current densities.

A two-electrode configuration is assembled for a supercapattery device using hydrothermally synthesized BaCuPO_4_ nanomaterial. Herein, active carbon (AC) corresponds to the -ve electrode, while the schematic diagram in Fig. 6a demonstrates the BaCuPO_4_ owing to the positive electrode. Necessarily, the electrolyte utilized in this scheme is 1 M KOH. All its electrochemical measurements are observed to show novelty in hydrothermal strategies for supercapattery devices. First, the optimized potential window is set as 0–1.6 V. Three comparative electrochemical investigations of CV, GCD, and cyclic stability were performed to investigate the unique presentation of the device. Before characterization, both electrode stability tests are performed within the prescribed window where CV comparison is presented, as shown in Fig. 6b. The operating potential (OP) for activated carbon was assigned to be −1 to 0 V, while for active material, BaCuPO_4_ was 0–0.6 V. The OP for supercapattery was 0–1.6 owing to the combined OP of AC and BaCuPO_4_. For electrochemical measurements, Fig. 6c shows CV analysis performed against various scan rate ranges of 5–100 mV s^−1^ owing to the same potential. The CV trend of the real device does not match the traditional behavior of carbonaceous and battery nature electrodes, indicating that the storage mechanism is severely caused by surface control and/or diffusion control processes. Physics behind the absence of a peak at more miniature potential reveals that the accumulation of charge occurred because of the adsorption process, while poor peaks appearing towards higher potentials is the sign of a diffusion-variant charge storage process.

Moreover, the CV results confirmed the device's stability as the optimum 100 mV s^−1^ scan rate and that the minimum scan rate gives the same pattern of CV curves. Furthermore, GCD evaluation was performed against different levels of current densities to explore device efficiency within the same potential window, as illustrated in Fig. 6d. The GCD curve behavior denotes non-triangular and non-humpy shapes, confirming that the charging/discharging mechanism was strictly owing to surface and diffusion control interactions.

**Fig. 7:**
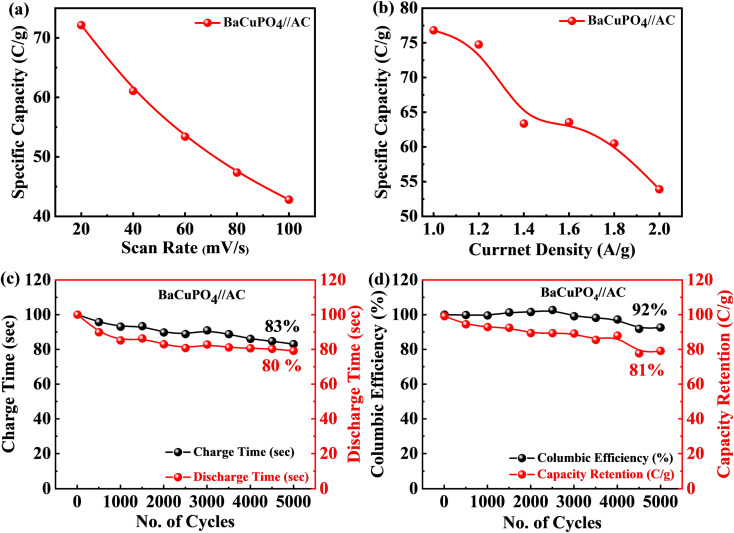
(a) Specific capacities against scan rates for supercapattery performance, (b) specific capacities as a function of current densities for supercapattery performance, (c) charging and discharging stability performance, and (d) cyclic stability efficiency of the device.

The specific capacity calculated at a minimum scan rate of 5 mV s^−1^ was 74 C g^−1^ using eqn (2) for the supercapattery device, as demonstrated in Fig. 7a. In addition, the optimum specific capacity of this ideal device evaluated through GCD using eqn (3) is 77 C g^−1^, corresponding to a current density of 1.0 A g^−1^, as illustrated in Fig. 7b. The retention uniqueness of the device was checked for continuous charging/discharging with 5000 consecutive cycles. The device performance shows the supercapattery nature for charging time efficiency as 83%, while discharging time efficiency is 80% at 5000 cycles, as depicted in Fig. 7c. A durability test was also performed to investigate the efficiency rate, significantly revealing that the novel device retained capacity retention of 81% and a coulombic efficiency of 92% caused by the 5000 cycling mechanism, as shown in Fig. 7d. A comparison of capacity retention and coulombic efficiency with previous studies is shown in Table 3.

**Fig. 8:**
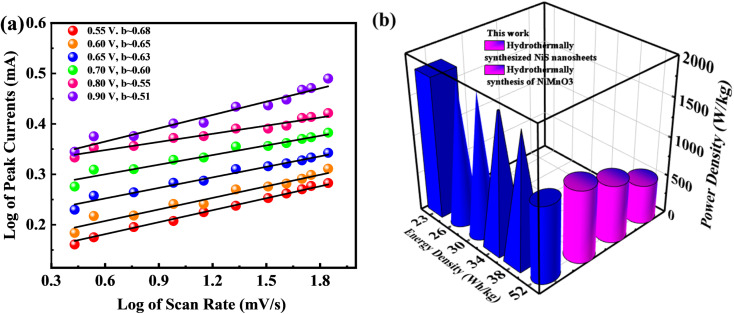
(a) Plots of log of peak currents against the log of scan rates revealing supercapattery performance. (b) High energy density and power density performance of the device compared to others.

Subsequently, Fig. 8a illustrates a graphical view of the log corresponding to peak currents displaying and the log attributing to scan rate while computing the *b*-fitting outcomes. The *b*-value is involved in estimating the efficiency of supercapattery charge storage capacity. Fig. 8b represents the probable *b*-value, as computed by following the slope of the graphical result sketched between the log of the log of scan rates and optimum currents. The *b*-value aforementioned discriminates among various devices, such as battery type, supercapacitor nature, and supercapattery-ranked materials. Under this classification, the storage device is regarded as battery-graded when the *b*-value level lies in the range of 0–0.5. Additionally, the supercapacitor *b*-value falls in the range of 0.8–1.0; and finally, the device becomes supercapattery nature because of the *b*-value lying in the range of 0.5–0.8.^50^

Furthermore, for energy storage strategies, two key parameters indicating energy density with power density are suggested as mandatory characteristics for analyzing its novelty. Consequently, energy density and power density are evaluated using the following equations:4
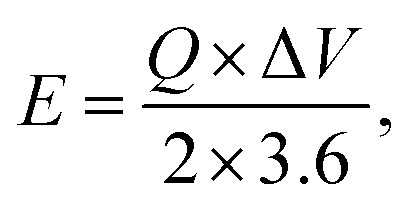
5
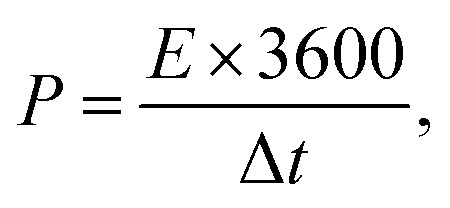
where *E* denotes energy density, *Q* denotes specific capacity, *V* denotes potential, *P* denotes power density, and Δ*t* denotes discharging time. In this respect, the evaluated energy and power density measurements were 52.13 W h kg^−1^ and 950 W kg^−1^, respectively, owing to the fabricated BaCuPO_4_//AC device. Further, the optimum power density valued at 4500 W h kg^−1^ was investigated, corresponding to 12 W h kg^−1^ of energy density. In addition, a comparative study of energy and power density with previous studies is demonstrated in Fig. 8b. Moreover, Table 1 was also added, representing a comparison of energy density with power density belonging to the supercapattery BaCuPO_4_//AC.

**Table 1:** Comparison of energy and power density of hydrothermally synthesized supercapattery BaCuPO_4_ with previous studies

Previous study	*E* (W h kg^−1^)	*P* (W kg^−1^)
Co_3_(PO_4_)_2_ device^51^	35.5	293.9
(NiCo(PO_4_)_3_//AC) device^52^	34.8	377
Co_3_(PO_4_)_2_/GF	52	847
Mn_3_(PO_4_)_2_ device^53^	11.7	1410
This work (hydrothermal)	52.13	950

**Table 2:** Comparison of *Q*_s_ of this work with previous studies

Literature	*Q*_s_ (mA h g^−1^)
(NiCo(PO_4_)_3_//AC) device^52^	86.4
Co_3_(PO_4_)_2_/GF^53^	57
Potassium Co–Ni phosphate^54^	34.7
Ni_3_(PO_4_)_2_–NbPO_4_/CNT^55^	208
This work: chemical precipitation, hydrothermal	620, 755

**Table 3:** Comparison of coulombic efficiency and capacity retention with previous studies

Literature	No. of cycles	Coulombic efficiency	Capacity retention
Co_3_ (PO_4_)_2_ (ref. 56)	5000	—	92.9%
CoS/Co_3_ (PO_4_)_2_ (ref. 57)	5000	—	95.10%
(Co_0.125_Cu_0.375_Mn_0.50_(PO_4_)_2_)^58^	2000	—	97.2%
Co_3_(PO_4_)_2_ (ref. 59)	10 000	—	87.2%
This work (hydrothermal)	5000	81%	92%

**Fig. 9:**
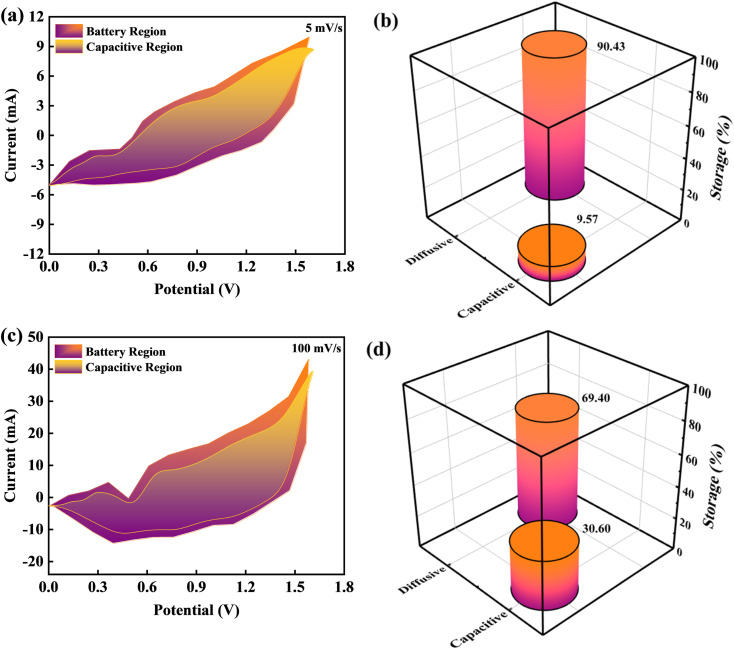
Dunn's model illustrating capacitive and diffusive portions at scan rates of (a) 5 mV s^−1^ and (c) 100 mV s^−1^, respectively. Bar chart depicting the capacitive and diffusive portions at scan rates of (b) 5 mV s^−1^ and (d) 100 mV s^−1^, respectively.

To investigate the capacitive and diffusive findings attributed to CV curves owing to BaCuPO_4_//AC device outcomes, Dunn's method was employed, as illustrated in Fig. 9. The CV curves of BaCuPO_4_//AC device revealed various redox peaks representing the capacitive and diffusive mechanisms. In this regard, the Dunns model was used for the deployment of CV curves to govern capacitive and diffusive parts.

6 *i*(*v*) = *k*_1_*v* + *k*_2_*v*^0.5^.

7
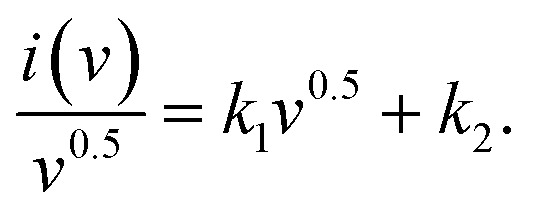


From eqn (6) and (7), *k*_1_*v* represents the capacitive part, while *k*_2_*v*^0.5^ represents the diffusive region. Further, *k*_1_*v* represents the capacitive current region associated with fast and reversible redox reactions occurring at interfacial contact between the electrolyte and electrode. Significantly, the current increases linearly, which shows the scan rate (*v*) relating to the slope of *k*_1_*v*, thereby exhibiting that the capacitive charge storing process has a proportionate relation with the scan rate. The *k*_2_*v*^0.5^ component reveals the diffusive part in the current region attributable to the ion diffusion associated with the electrode matrix. In this context, the square root of *v*^0.5^ represents the optimal response observed in diffusion-limited systems. Fig. 9a–c depicts the capacitive and diffusive segments of the CV plots for the BaCuPO_4_//AC device at scan rates of 5 and 100 mV s^−1^, respectively. Furthermore, the bar chart clearly distinguishes between the capacitive and diffusive regions, as depicted in Fig. 9b–d. The graphs show that the diffusive component dominates at the lower scan rate, demonstrating an effectiveness of 90.74% in the diffusive region. Hence, this proved the enhanced efficiency of the diffusive electrodes, which possessed much time to ample the faradaic reactions at a lower scan. In contrast, as the scan rate increases, the capacitive contribution appears dominant. Consequently, the BaCuPO_4_//AC device demonstrated an enhanced capacitive efficiency of 30.60% at the elevated scan rate. Intriguingly, this exceptional capacity retention, improved coulombic efficiency, impressive energy density, and power density exhibited by the BaCuPO_4_//AC device highlight its distinctive potential as a promising candidate for energy storage devices and hydrogen evolution reactions.

### 5.2 Hydrogen evolution reaction (HER)

**Fig. 10:**
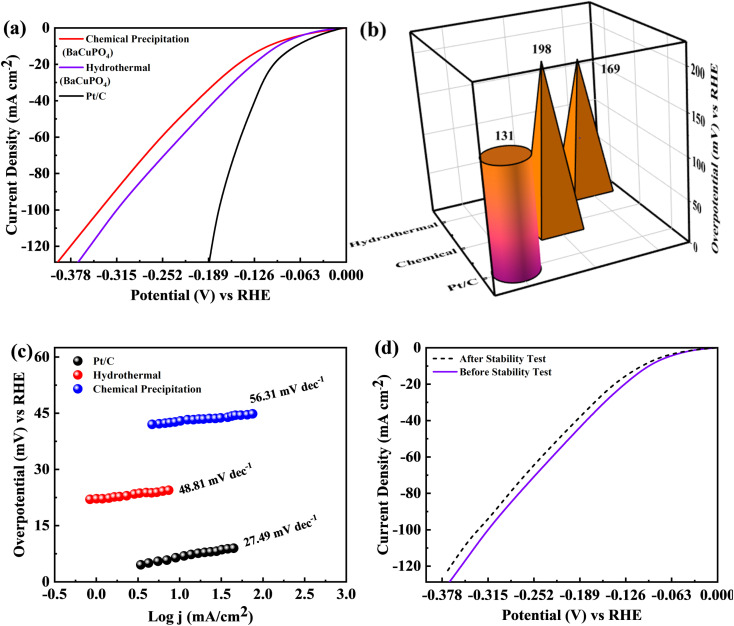
(a) Polarization curves for Pt/C, hydrothermal and chemical precipitation synthesized BaCuPO_4_ at 2 mV s^−1^. (b) Overpotential *vs.* RHE outcomes for Pt/C, hydrothermal and chemical precipitation. (c) Tefel slope for Pt/C, hydrothermal and chemical precipitation synthesized BaCuPO_4_. (d) LSV curve before and after 5000 CV cycles.

The BaCuPO_4_ synthesized through both methods was employed as a self-supported electrocatalyst for HER. The polarisation behaviors of the catalysts, *i.e.*, hydrothermal BaCuPO_4_ and precipitated BaCuPO_4_ devices using 1 M KOH solution at a scan rate of 2 mV s^−1^ exhibit HER performance efficiency, as illustrated in Fig. 10a. Consequently, the hydrothermal BaCuPO_4_ device and the standard catalyst Pt/C perform exceptional HER activity. Moreover, by exploring the lower overpotential of 131 mV, the Pt/C showed outstanding performance, as shown in Fig. 10b. Therefore, their excellent results prove the great effectiveness and activeness as a novel electrocatalyst of hydrothermal BaCuPO_4_ device while accelerating the HER mechanism compared to traditional catalysts. Additionally, the efficiency gap attributed to Pt/C and BaCuPO_4_ is significantly noticeable through superior *j*_s_ resulting in 200 mA cm^−2^. Hence, the hydrothermally developed BaCuPO_4_ needs a moderate overpotential of only 169 mV, while chemically synthesized BaCuPO_4_ needs an overpotential of 198 mV. Based on these significant results, the BaCuPO_4_ cell proved to be a leading catalyst for commercial HER catalysts. The role of the BaCuPO_4_ cell, which is a superior and low-cost catalyst, is additionally reinforced by the vast comparative data presented in Table 4, thereby highlighting its potential for revolutionizing the discipline owing to electrocatalysis and, in turn, opening the new door to improve environmentally friendly HER performance. In this context, the Tafel graphs attributed to the electrodes are illustrated in Fig. 10c. Remarkably, the hydrothermally synthesized BaCuPO_4_ possessed a Tafel slope with 48.81 mV dec^−1^ values, and BaCuPO_4_ synthesized using the chemical precipitation method showed a Tafel slope of 56.31 mV dec^−1^. In this way, it exhibits the fast activation of the Volmer–Heyrovsky mechanism, which interestingly fast-tracks the reaction rates. Hence, the results indicate the great potential of BaCuPO_4_ for a highly active catalyst for HER implementations by adding significant evidence in the form of its rapid and effective reaction kinetics. Moreover, the HER proficiency of BaCuPO_4_ reveals strange stability, showing minimal current loss by completing 5000 cycles (Fig. 10d). Consequently, this establishes how the BaCuPO_4_ becomes robust, delivering reliable performance over a prolonged functioning duration. Finally, the findings aforementioned for the position of the BaCuPO_4_ cell explore it as a promising and reliable catalyst for HER applications, offering the potential for charge transfer proficiencies and excessive stability with enduring operating efficiency, particularly in difficult surroundings.

**Table 4:** HER performance comparison with previous studies

Literature	Overpotential (mV)	Tefel slope (mV dec^−1^)
Ni_3_(PO_4_)_2_–NbPO_4_/CNT^55^	133	57.7
MoS_2_ quantum dots^60^	200	65
NiFe^61^	230	33
(1T and 2H) MoSe_2_ (ref. 62)	175	58
Ni-BTC^63^	53	62
Cu_2_O nanorods^64^	184	106
This work: chemical precipitation, hydrothermal	198, 169	56.31, 48.81

## 6. Conclusions

This work demonstrates a significant effect of synthetic strategy on the electrochemical presentation of BaCuPO_4_. For this purpose, BaCuPO_4_ was synthesized using hydrothermal and chemical precipitation methods. Crystal structural and morphological examinations performed using XRD and SEM demonstrated that NSs synthesized using the hydrothermal method were more suitable for energy storage devices than those synthesized using the chemical precipitation technique. In the three cell assemblies, the optimum specific capacity of 764.4 C g^−1^ was evaluated for hydrothermally synthesized BaCuPO_4_, which was far better than the chemical precipitation method. The supercapattery was further formed with hydrothermally produced BaCuPO_4_ acting in an anodic nature, whereas activated carbon behaved in a catholic nature. This supercapattery device exhibits an energy density of 52.13 W h kg^−1^. Thus, the maximum power obtained from BaCuPO_4_//AC was 4500 W kg^−1^. The capacity retention and coulombic efficiency were 92% and 81% for BaCuPO_4_//AC after 5000 cycles, respectively. Additionally, the hydrothermally prepared BaCuPO_4_ showed an overpotential of 169 mV and a Tefel slope of 48.81 mV dec^−1^ compared to the chemical co-precipitation results. This study reveals that the hydrothermal method is more beneficial for supercapacitor applications and HER than the chemical precipitation method.

## Conflicts of interest

There are no conflicts to declare.

## Supplementary Material

RA-013-D3RA07596F-s001
