# Supplementary material for: Analyzing synthesis routes for BaCuPO4: implications for hydrogen evolution and supercapattery performance
Source: RSC Adv. 2023 Dec 5;13(50):35468–80. doi: 10.1039/d3ra07596f (PMC10696637; doi:10.1039/d3ra07596f)
Supplement: RA-013-D3RA07596F-s001 [file RA-013-D3RA07596F-s001.pdf]

Electronic Supplementary Material (ESI) for RSC Advances.
This journal is © The Royal Society of Chemistry 2023

Analyzing Synthesis Routes for BaCuPO4: Implications for Hydrogen Evolution and Supercapattery Performance"

Sarfraz Ali[1], Haseebul Hassan[1], Muhammad Waqas Iqbal[1*], Amir Muhammad Afzal[1], Mohammed A. Amin[2], A. Alhadrami[2], Nawal D. Alqarni[3], Ehtisham Umar[4],

[1]Department of Physics, Riphah International University, Campus Lahore, Pakistan

[2]*Department of Chemistry, College of Science, Taif University, P.O. Box 11099, Taif*

[3]*Department of Chemistry, College of Science, University of Bisha, Bisha, 61922, Saudi Arabia*

[4]*Department of Physics, Government College University Lahore, 54000, Punjab, Pakistan*

E-mail: waqas.iqbal@riphah.edu.pk

Supplementary Section:

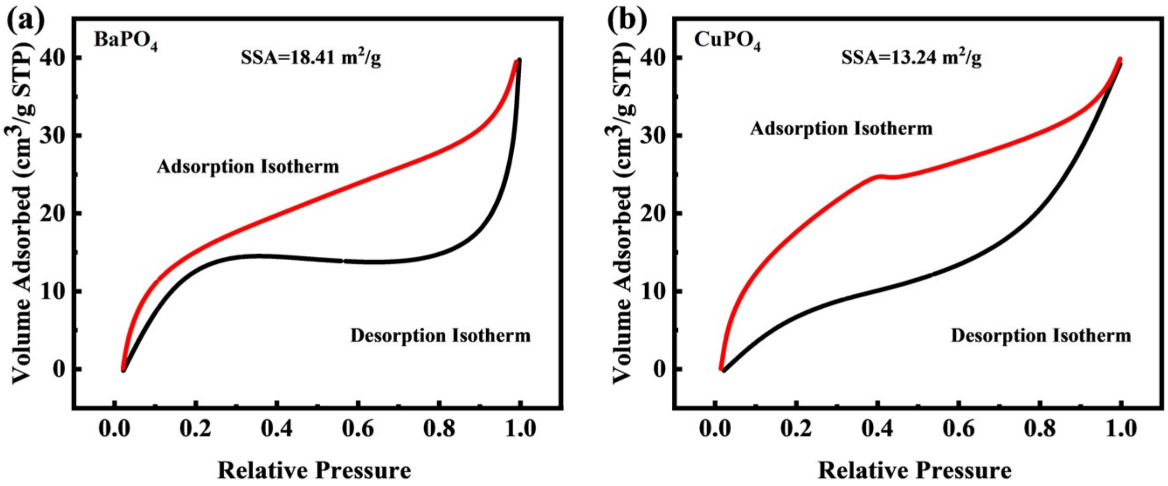

Fig. S1 BET isotherm of $BaPO_4$ and $CuPO_4$ nanocomposites.

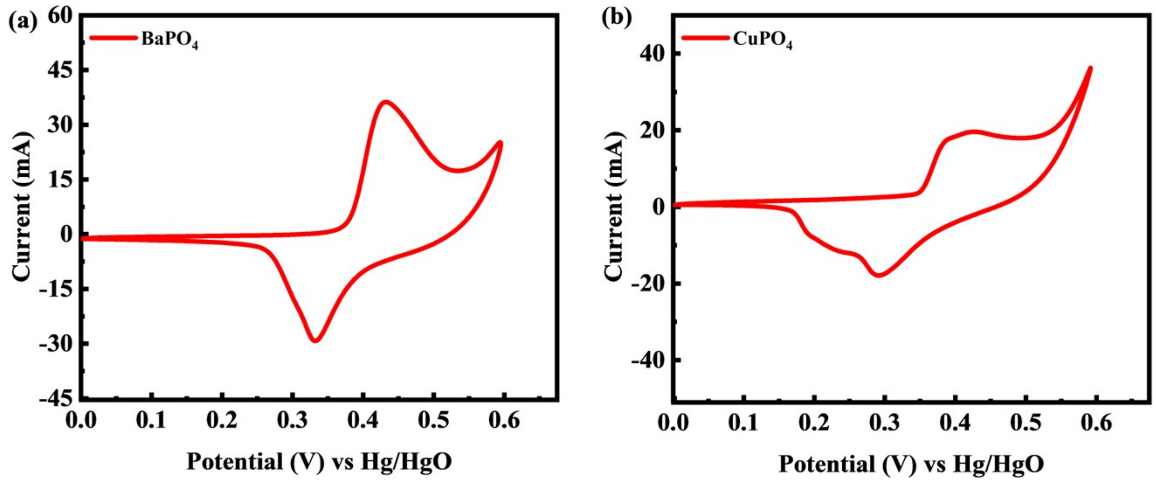

Fig S2 Cyclic voltammetry of $BaPO_4$ and $CuPO_4$ nanocomposites at 3 mV/s scan rat.

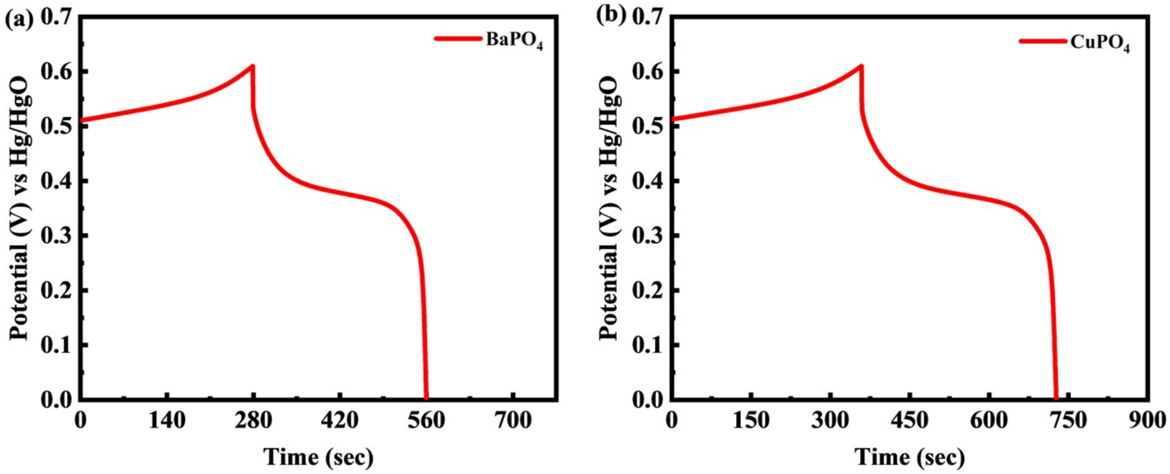

Fig S3 Galvanostatic charge-discharge of $BaPO_4$ and $CuPO_4$ nanocomposites.
